# IL‐11—An aging‐related cytokine with opportunities for regulating hematopoiesis

**DOI:** 10.1002/hem3.70050

**Published:** 2024-11-19

**Authors:** David G. Kent

**Affiliations:** ^1^ Department of Biology, Centre for Blood Research, York Biomedical Research Institute University of York York UK

Cytokines have long been known as chemical messengers that act upon cells in the blood system to induce proliferation and response to disease. Studying them at scale in a tissue context has been challenging due to functional redundancy and pleiotropic effects, but they remain an area of active investigation for a wide range of groups, especially now that new approaches are emerging to studying cytokine signaling dynamics at the single molecule level.^1^, ^2^, ^3^ This summer, a huge study on the proinflammatory cytokine Interleukin 11 (IL‐11) emerged in *Nature* magazine from the group of Stuart Cook—the paper *Inhibition of IL‐11 signaling extends mammalian healthspan and lifespan* ^4^ dropped into the hyperactive aging research community and caused quite a stir.

The headline statement: “Genetic deletion of *Il11* extended the lives of mice of both sexes, by 24.9% on average” caught the research world's attention. This was impressively followed by a series of studies that involved treating mice with an anti‐IL‐11 antibody from middle age (75 weeks) until death where the researchers observed another impressive increase in lifespan (>20%), strongly suggesting that early life events do not irrevocably sentence an organism to an early death. Simple in design and elegant in execution, the study details the genetic and pharmacological modulation of aging in both sexes. This positions the paper as one of the few that demonstrates a clear extension of lifespan through the removal of a single gene—one of the earliest examples of which is the *daf‐2* c.elegans mutants^5^ that have an extended lifespan. The authors go on to detail a wide range of metabolic and pathway profiling and imply that the metabolic, proinflammatory, and profibrotic roles of IL‐11 are the mechanistic drivers of aging through the ERK‐mTORC1 and JAK/STAT signaling pathways. This also suggests that JAK inhibitors, metformin, rapamycin, and so forth might have antiaging and antifibrotic roles as well, but the authors note that some of these current therapies struggle with on‐ and off‐target toxicities that an anti‐IL‐11 therapy might not have. Indeed, an early‐stage clinical trial is already underway using anti‐IL‐11 for the treatment of fibro‐inflammatory diseases.

IL‐11 is no stranger to stem cell and hematopoiesis research. It is one of a handful of critical molecules that interact with the glycoprotein 130 (gp130) family for signal transduction, in the good company of leukemia inhibitory factor (LIF) and interleukin 6 (IL‐6) among others. These molecules have long been studied in stem cell systems (e.g., LIF in mouse embryonic stem cells and ciliary neurotrophic factor [CNTF] in neural stem cells) and have also been among the key regulators of hematopoietic stem cell (HSC) self‐renewal in the form of IL‐6 and IL‐11. Early studies highlighted IL‐11's partnership with the stem cell factor (SCF) in maintaining self‐renewal in HSC expansion cultures,^6^, ^7^ but equally, IL‐11 has been shown to be dispensable for the more recent and better performing PVA‐based cultures systems described by Yamazaki and colleagues which used thrombopoietin (TPO) and SCF to achieve HSC expansion.^8^ Interestingly, however, IL‐11 on its own has been recently shown to maintain HSCs in a hibernating state outside the body in both the mouse and human settings.^9^, ^10^ HSCs were maintained as single cells in the absence of a physical niche and retained their full functional potential in transplantation assays compared to freshly isolated HSCs. Taken in the context of the new findings from the Cook group, it now becomes critical to work out the relationship of IL‐11 signaling in aging HSCs—what would be blocking this key regulator during HSC aging and how would it influence the acquisition of clonal hematopoiesis, accumulation of inflammatory cytokines, or leukemia development? Perhaps, one of the most interesting areas to consider is that the hibernation cultures are fully reversible with respect to HSC function whereas the Cook study draws the linkage to IL‐11 driving an irreversible senescent state.

In any event, it almost seems certain that the anti‐IL‐11 therapy described in the paper will be explored widely for its potential application in humans. It will be incredibly interesting to understand the changes in the blood system, and more specifically on HSCs and inflammation‐induced changes with aging. A 25% extended lifespan from blocking a single molecule seems too good to be true from a direct translation to the human point of view, but every little bit helps and there is almost certainly some fascinating science to unearth.

## AUTHOR CONTRIBUTIONS

David G. Kent is the sole author and wrote the article.

## CONFLICT OF INTEREST STATEMENT

The author declares no conflict of interest.

## FUNDING

The Kent lab is funded by the UK Medical Research Council, the Bill and Melinda Gates Foundation, Blood Cancer UK, the Rosetrees Trust, and Cancer Research UK.

## Data Availability

Data sharing is not applicable to this article as no data sets were generated or analyzed during the current study.
